# Comprehensive analysis of cuproptosis-related genes on bladder cancer prognosis, tumor microenvironment invasion, and drug sensitivity

**DOI:** 10.3389/fonc.2023.1116305

**Published:** 2023-02-21

**Authors:** Honglei Wang, Jinqiao Li, Xiaolin Zi, Xueli Yuan

**Affiliations:** ^1^ Department of Urology, Harbin Medical University Cancer Hospital, Harbin, China; ^2^ Heilongjiang Key Laboratory of Scientific Research in Urology, Fourth Hospital of Harbin Medical University, Harbin, China; ^3^ National Health Commission (NHC) Key Laboratory of Molecular Probes and Targeted Diagnosis and Therapy, Harbin Medical University, Harbin, China; ^4^ Department of Medical Oncology, Fourth Hospital of Harbin Medical University, Harbin, China

**Keywords:** bladder cancer, prognosis, cuproptosis, tumor microenvironment, therapy

## Abstract

Cuproptosis, a newly discovered form of programmed cell death, plays a vital role in the occurrence and development of tumors. However, the role of cuproptosis in the bladder cancer tumor microenvironment remains unclear. In this study, we developed a method for predicting the prognostic outcomes and guiding the treatment selection for patients with bladder cancer. We obtained 1001 samples and survival data points from The Cancer Genome Atlas database and Gene Expression Omnibus database. Using cuproptosis-related genes (CRGs) identified in previous studies, we analyzed CRG transcriptional changes and identified two molecular subtypes, namely high- and low-risk patients. The prognostic features of eight genes (*PDGFRB*, *COMP*, *GREM1*, *FRRS1*, *SDHD*, *RARRES2*, *CRTAC1*, and *HMGCS2*) were determined. The CRG molecular typing and risk scores were correlated with clinicopathological features, prognosis, tumor microenvironment cell infiltration characteristics, immune checkpoint activation, mutation burden, and chemotherapy drug sensitivity. Additionally, we constructed an accurate nomogram to improve the clinical applicability of the CRG_score. qRT-PCR was used to detect the expression levels of eight genes in bladder cancer tissues, and the results were consistent with the predicted results. These findings may help us to understand the role of cuproptosis in cancer and provide new directions for the design of personalized treatment and prediction of survival outcomes in patients with bladder cancer.

## 1 Introduction

Bladder cancer (BC) is one of the most common solid tumors worldwide. It is the seventh most common malignant tumor in men and the tenth most common in women, with an estimated 550,000 new cases and 20,000 deaths yearly (1–4). BC is divided into urothelial carcinoma, squamous carcinoma, and adenocarcinoma. Bladder urothelial carcinoma is the most common subtype, accounting for more than 90% of cases (4, 5). Currently, the main treatment method for BC is surgery combined with other therapies; however, due to its high recurrence rate and frequent metastasis, the patient prognosis remains unsatisfactory (5, 6). Molecular typing by gene analysis has become a focus because BC occurrence and development is a complex pathological process (7). To improve the early management and survival rate of patients with BC, it is critical to find new biomarkers and explore new molecular subtypes to predict patient prognosis and drug treatment response.

Copper is an essential trace element for the human body and an important cofactor in many physiological processes, such as mitochondrial respiration and in antioxidants (8, 9). Abnormal copper levels are associated with a variety of diseases (10–12), including high levels in malignant tumors (13, 14). In patients with BC, copper has been found to be abnormal both in the serum and cancer tissues. Furthermore, copper enhances angiogenesis and is involved in the occurrence and development of BC (15, 16). Recent studies have identified a new and non-apoptotic form of programmed cell death induced by copper called cuproptosis (13, 17, 18). In a study published by Tsvetkov et al. (2), intracellular copper was found to bind to the stellated component of the TCA cycle (19, 20), which aggregates mitochondrial lipidated proteins and leads to reduced stability of iron-sulfur clustering (21, 22), leading to this unique type of cell death. Additionally, this report also identifies a series of key genes for cuproptosis.

However, this new form of cell death requires further investigation in BC development. Identifying the characteristics of cuproptosis-related genes in BC and patient subtyping will help to improve understanding of BC occurrence, development, and mechanisms, provide personalized treatment plans, and predict treatment response.

In this study, we combined BC sample transcript data from The Cancer Genome Atlas (TCGA) and Gene Expression Omnibus (GEO) databases to classify patients into two distinct molecular subtypes based on the differentially expressed cuproptosis-related genes. These groups showed different prognoses and immune characteristics. We also established a cuproptosis scoring system for BC, which can help evaluate the cuproptosis pathway in tumors and predict the prognosis of patients, the effects of immunotherapy, and personalized treatment plans.

## 2 Materials and methods

### 2.1 Acquisition of data

TCGA (https://portal.gdc.cancer.gov/) and GEO databases (https://www.ncbi.nlm.nih.gov/geo/) were used to download BC gene expression data and corresponding clinical information. The fragments per kilobase million value of the TCGA-BLCA cohort was converted into transcripts per million, which was merged with three GEO cohorts (GSE13507, GSE31684, and GSE32894). Patient data with missing survival information were removed. The batch effect was eliminated using the “ComBat” algorithm in the “sva” R program (23). In total, 1001 patients were included in the analysis. Somatic mutation data and copy number variation data were downloaded from the TCGA database. Thirteen cuproptosis-related genes (CRGs) were obtained from these data.

### 2.2 Consensus cluster analysis of CRGs

An unsupervised consensus cluster analysis was performed on the 13 CRGs using the “ConsensusClusterPlus” R package (24) to classify the patients into different molecular subtypes. The number of clusters was set from 2 to 9, and selection was based on the following criteria: the concordant product distribution function curve had a small slope of decline, there was no small sample size group, and clustering showed high intra-group correlation and low inter-group correlation. Principal component analysis was used to reduce the dimensions and distinguish different subtypes of information.

### 2.3 Relationship between molecular subtypes, clinical features, and prognosis of patients with BC

The R package “beeswarm” (25) was used to compare the clinical value of the two subtypes. The correlation between clinical parameters age (≤ 65 years or > 65 years), sex (female or male), tumor stage (T1, T2, T3, or T4), grade (high or low grade), and the prognosis was evaluated and illustrated with heatmaps.

### 2.4 Identification and functional analysis of DEG

Using the “limma” R package (26), we set the filter criteria as log(Fold Change) > 1 and adjusted the p-value to be < 0.05 to obtain differentially expressed genes (DEGs) between different cuproptosis subtypes. These DEGs were then subjected to functional enrichment analysis using Gene Ontology and Kyoto Encyclopedia of Genes and Genomes analyses in the “clusterProfiler” R package (27).

### 2.5 Construction of the cuproptosis-related prognostic score model

First, univariate COX regression analysis was performed on the DEGs between the two subtypes, and the genes related to prognosis were screened. Unsupervised consensus cluster analysis was performed using the “ConsensusClusterPlus” R package (24) to classify the BC patients in the combined cohort into different gene subtypes. Next, the patients were randomly divided into either the training or testing groups. The optimal results were obtained by LASSO regression analysis of the training group data using the R package “glmnet” (28). A “multiCox” analysis was performed to obtain eight central genes and correlation coefficients. Furthermore, a prognostic model of cuproptosis was established, named CRG_score, where CRG_score = (expression of each gene × correlation coefficient). Based on the median value of the risk score, patients in the training group were divided into high- and low-risk groups. Kaplan–Meier survival analysis was performed, and a receiver operating characteristic curve (ROC) was constructed. To verify the predictive ability of the model, we evaluated its prognosis, sensitivity, and specificity in the testing group. In addition, the “rms” package was used to create a prognostic nomogram and a calibration map.

### 2.6 Tumor microenvironment analysis of CRG_score

To evaluate the relationship between the CRG_score and TME, the “CIBERSORT” R package (29) was used to evaluate the abundance of immune cells infiltrating tumors in high- and low-risk samples. The correlation between the score and infiltrating cell abundance was analyzed using linear analysis and a heatmap. Simultaneously, the immune cells, matrix, and ESTIMATE scores of patients with BC were calculated using the ESTIMATE algorithm. In addition, we analyzed the differences in the expression of immune test genes in the different CRG_score groups.The single-cell tumor microenvironment expression of cuproptosis-related genes in the tumor microenvironment was analyzed using the TISCH database(http://tisch.comp-genomics.org/).

### 2.7 Drug sensitivity analysis

To explore the sensitivity of BC high- and low-risk groups to chemotherapies, we calculated the half-maximal inhibitory concentration (IC50) values of four commonly used BC drugs using the “pRRophetic” R package (30) based on the Cancer Drug Sensitivity Genomics database. Drug sensitivity was positively correlated with IC50 values.

### 2.8 Tissue sample collection

This study was approved by the Ethics Committee of the Fourth Affiliated Hospital of Harbin Medical University. All patients provided signed informed consent before participating in the study. Thirty BC tissue samples and 30 normal BC epithelial tissue samples were collected from patients undergoing surgical resection at the Department of Urology, Fourth Affiliated Hospital of Harbin Medical University. The obtained tissue samples were snap-frozen in liquid nitrogen and stored at −80°C.

### 2.9 RNA extraction and quantitative reverse transcription-polymerase chain reaction

RNA was extracted from the tissues using TRIzol reagent (Invitrogen, Carlsbad, CA, USA) according to the manufacturer’s instructions. The isolated RNA was reverse transcribed into cDNA using a reverse transcription kit (Takara Bio, Iapan). qRT-PCR was performed using the SYBR Green PCR Master Mix (Takara Bio). The following thermal cycling conditions were used: amplification reactions were performed at 95°C for 1 min and 95°C for 15 min, followed by 40 cycles at 95°C for 15 s and at 52°C for 15 s. All qRT-PCR reactions were performed in duplicates. The primer sequences used are listed in **Supplementary Table 2**. The relative quantitative method 2−ΔCT was used to analyze the expression data.

### 2.10 Statistical analyses

All statistical analyses were performed using the R software (version 4.0.5). Statistical significance was set at P < 0.05. In all figures: ∗ < 0.05, ∗∗ < 0.01, ∗∗∗ < 0.001.

## 3 Results

### 3.1 Genetic and transcriptional alterations of CRGs in BC

**Figure 1:**
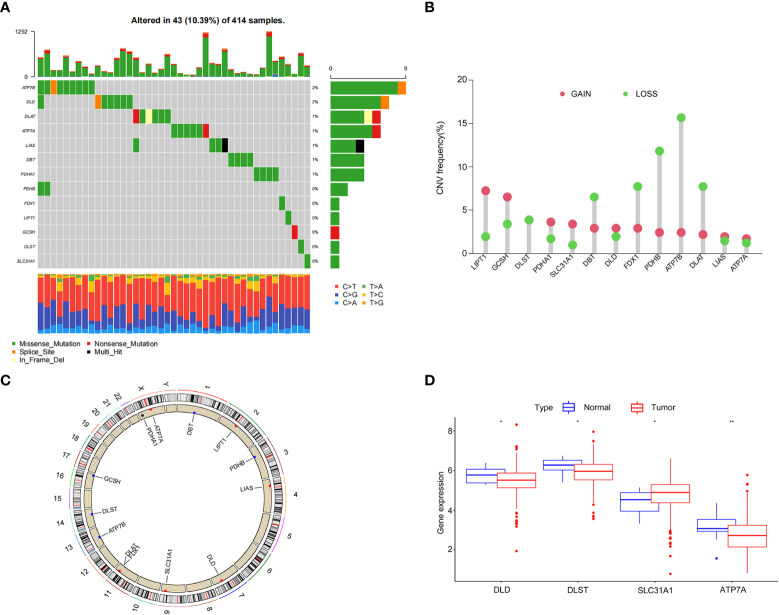
Genetic and transcriptional alterations of cuproptosis-related genes in bladder cancer. **(A)** Frequency of cuproptosis-related gene (CRG) somatic mutations in patients with bladder cancer in The Cancer Genome Atlas cohort. **(B)** Copy number variations (CNVs) in CRGs. **(C)** The chromosomal positions of CRGs with CNV. **(D)** Differential expression of CRGs in normal bladder tissue and bladder cancer tissue. *P < 0.05, **P < 0.01.

We analyzed the somatic mutation rates of 13 CRGs in the TCGA-BLCA cohort and found 43 mutations (10.39%) in the BC samples. The highest mutation frequency was observed in *ATP7B* (**Figure 1A**). However, we noticed that all CRGs had copy number variation (CNV) changes, among which *ATP7B, PDHB, FDX1*, and *DLATD* had the most significant decrease in copy number, whereas *LIPT1* and *GCSH* had a significant increase in copy number (**Figure 1B**). The chromosomal positions of CRGs with CNV changes are shown in **Figure 1C**.

Next, we analyzed the differences in CRG expression between tumor and normal tissues in the TCGA-BLCA cohort. Significant differences were observed in *DLD, DLST, SLC31A1*, and *ATP7A* expression, which was partially consistent with the CNV (**Figure 1D**). However, the expression of CRGs with a higher frequency of CNVs did not differ between BC and normal tissues. These results showed that there were differences in the genetics and expression levels of CRGs between BC samples and normal tissues, suggesting a potential role for CRGs in BC.

### 3.2 Identification of cuproptosis subtypes in BC

**Figure 2:**
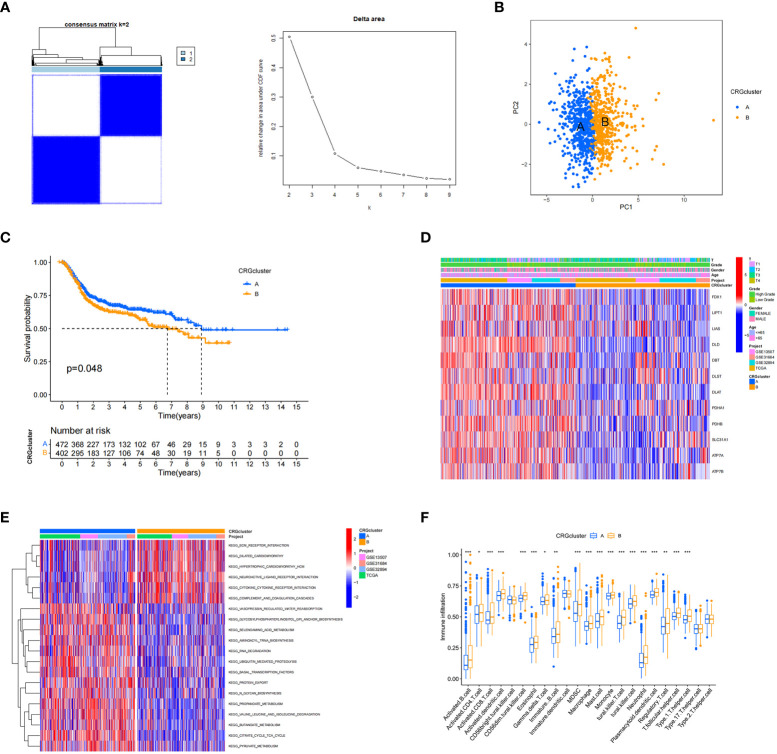
Cuproptosis-related gene subtypes and clinicopathological and biological characteristics of two different subtype samples partitioned by consistent clustering. **(A)** Consensus matrix heatmap defining two clusters (k = 2) and their associated regions. **(B)** Principal component analysis showing a significant separation of transcriptional profiles between the two subtypes. **(C)** Kaplan–Meier survival analysis curves of the two subtypes. **(D)** Heatmap of clinicopathological features between cuproptosis-related gene (CRG) subtypes. **(E)** Gene set variation analysis of biological pathways between CRG subtypes, where red and blue represent activated and inhibitory pathways, respectively. **(F)** Differences in immune cell infiltration in the two subtypes. *P < 0.05, **P < 0.01, ***P < 0.001

A total of 1001 patients from three eligible BC cohorts (TCGA dataset, GSE13507, GSE31684, and GSE32894) were integrated for further analysis. Using the expression levels of 13 CRGs, the patients were divided into K groups (k = 2–9). Two molecular subtypes (k = 2) were optimal (**Figure 2A**). Principal component analysis revealed a significant separation between the transcriptional profiles of cuproptosis genes of the two subtypes (**Figure 2B**). Kaplan–Meier survival analysis was performed, and the results are shown in **Figure 2C**. We also analyzed and compared the patient clinicopathological features (**Figure 2D**). In addition, GSCV enrichment analysis was performed. We found that subtype A is mainly enriched in 14 pathways related to metabolisms, such as amino acid, propionate, and pyruvate metabolism. In subtype B, we found significant enrichment in pathways involving the extracellular matrix receptor, neuroactive ligand-receptor, and cytokine-receptor interactions, which are related to carcinogenesis and metastasis (**Figure 2E**). Next, we investigated the differences in TME immune infiltration. We identified 19 immune cells that were significantly different in abundance between the two subtypes **Figure 2F**. In addition to T helper cells, two cell types showed more infiltration in subtype A, and other immune cells, including activated B cells, CD4^+^ T cells, CD8^+^ T cells, dendritic cells, immature B cells, regulatory T cells, and ten other immune cells were significantly more abundant in subtype B.

### 3.3 Identification of gene subtypes based on differentially expressed genes

**Figure 3:**
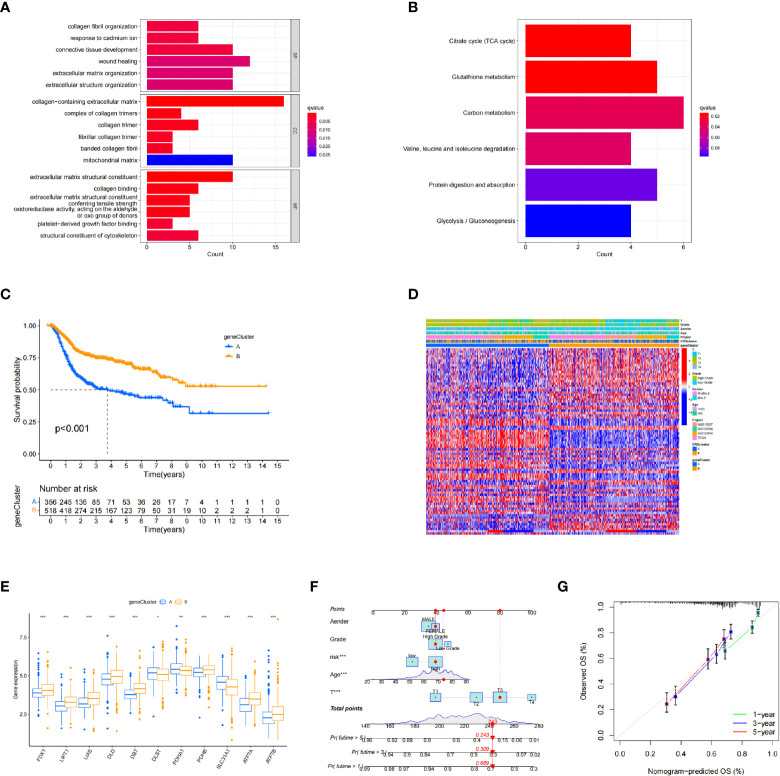
Identification of differentially expressed gene subtypes. **(A, B)** Gene Ontology and Kyoto Encyclopedia of Genes and Genomes enrichment analysis. **(C)** Kaplan–Meier survival analysis curves of the two subtypes. **(D)** Clinical case characteristics of differentially expressed gene subtypes. **(E)** Differences in immune cell infiltrates in the two subtypes. **(F, G)** Nomogram and calibration curve for bladder cancer patients using the CRG_score. *P < 0.05, **P < 0.01, ***P < 0.001

Next, we explored the biological differences between the cuproptosis subtypes in BC. First, 126 DEGs associated with cuproptosis subtypes were identified. Gene Ontology analysis showed that DEGs were significantly enriched in extracellular matrix-related biological processes (**Figure 3A**). Kyoto Encyclopedia of Genes and Genomes enrichment analysis showed that these DEGs were enriched in collagen fibril organization, connective tissue development, wound healing, extracellular matrix organization, and extracellular structure organization (**Figure 3B**). Thus, cuproptosis plays an important role in processes related to tumor invasion and metastasis through reordering the extracellular matrix. Univariate COX regression analysis identified 82 genes associated with prognosis. Using these prognostic DEGs, we divided the patients into two genotypes (type A and type B) using a consensus clustering algorithm, which showed that subtype A had significantly lower overall survival than subtype B (**Figure 3C**). The heatmap in **Figure 3D** depicts the correlation between clinical data and genotyping. The two gene subtypes showed significant differences in CRG expression levels, which was consistent with the cuproptosis pattern (**Figure 3E**).

### 3.4 Construction of a cuproptosis-related model

**Figure 4:**
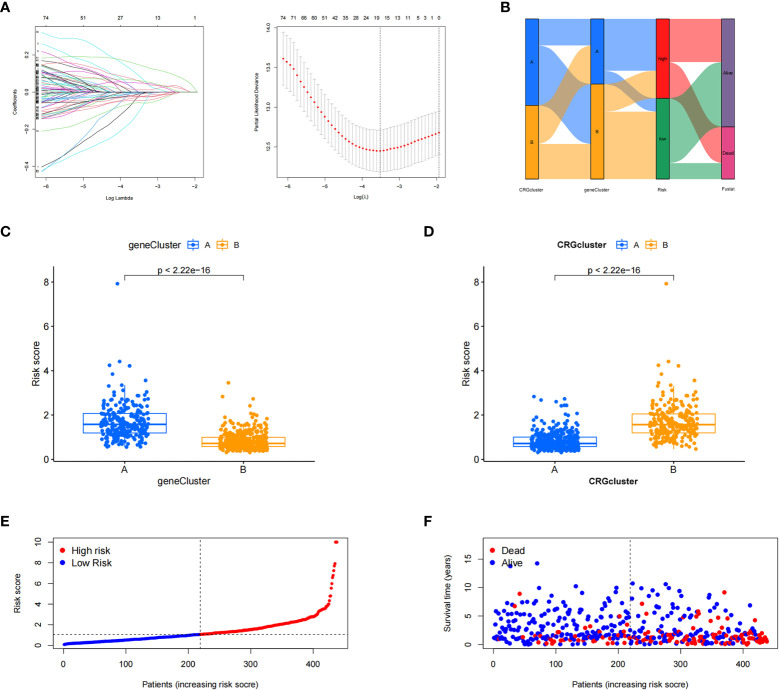
Construction of the cuproptosis-related gene scores. **(A)** Using the Trial group samples to find the best fit of LASSO regression. **(B)** Alluvial plot of the distribution of cuproptosis-related gene (CRG) subtypes and genotypes in different risk groups. **(C)** Differences in risk scores between genotypes. **(D)** Differences in risk scores between CRG subtypes. **(E, F)** Ordinal point and scatter plots of CRG risk score distribution and patient survival.

The patient samples were randomly divided into training (n = 438) and test (n = 437) groups at a ratio of 1:1. First, from the 13 prognostic DEGs, the best fit of LASSO regression (**Figures 4A, B**) and multivariate COX regression analysis was used to select eight genes. Among them were the high-risk genes *PDGFRB, COMP, GREM1*, and *FRRS1* and the low-risk genes *SDHD*, *RARRES2*, *CRTAC1*, and *HMGCS2*. Next, we constructed the prognostic features, and the CRG_score was calculated. The patient distribution in the cuproptosis subtypes, genotypes, and two prognostic risk score subtypes is shown in a Sankey diagram (**Figure 4C**). We also explored the relationship between CRC_scores and cuproptosis subtypes A and B and genotypes A and B. The CRG_score was significantly higher in subtype A than in subtype B, suggesting that a low CRG_score may be related to extracellular matrix-related features (**Figure 4D**). We also found that an increase in the CRG_score resulted in a decrease in survival time and an increase in mortality (**Figures 4E, F**).

### 3.5 Validation of the cuproptosis-related model

**Figure 5:**
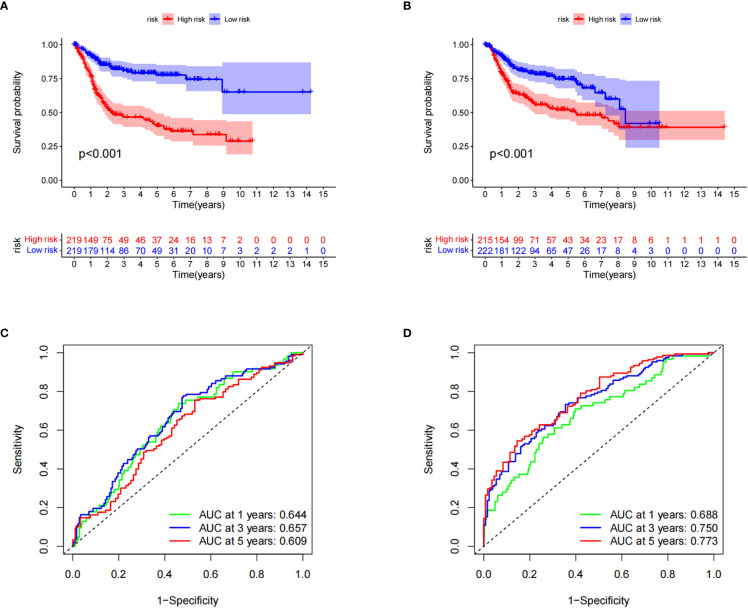
Verification of cuproptosis-related gene scores. **(A)** Kaplan–Meier survival analysis curves of the two groups in the training set. **(B)** Kaplan–Meier survival analysis curves of the two patient groups in the test set. **(C)** Receiver operating characteristic curves for predicting the sensitivity and specificity of 1-, 3-, and 5-year survival rates using the cuproptosis-related gene score (CRG_score) in the training set. **(D)** Receiver operating curves for the sensitivity and specificity of CRG_score in predicting 1-, 3-, and 5-year survival in the test set.

Kaplan–Meier survival curve analysis and log-rank tests showed that in both the training and testing groups, patients in the high-risk group had significantly lower survival rates than those in the low-risk group (**Figures 5A, B**). At the same time, we also examined the sensitivity and specificity of CRG_score. The ROC results showed that the area under the curve of the 1-, 3-, and 5-year survival times of the training group was 0.644, 0.657, and 0.609, respectively, and the area under the curve of the 1-, 3-, and 5-year survival time of the testing group was 0.688, 0.750, and 0.773, respectively, indicating that the CRG_score can robustly and reliably predict the prognosis of patients with BC (**Figures 5C, D**).

### 3.6 Construction of the nomogram

A nomogram is a tool that can be easily applied clinically to predict tumor prognosis. Due to the inconvenience of direct application of the CRG_score, we created a nomogram of BC based on the CRG_score and independent risk factors (patient sex and age, cancer grade, and tumor stage) (**Figure 3F**). In addition, we assessed nomogram accuracy using calibration curves, which showed high agreement between the clinical observations and nomogram predictions for 1-, 3-, and 5-year overall survival probabilities (**Figure 3G**).

### 3.7 Evaluation of TME and checkpoints between the two risk groups

**Figure 6:**
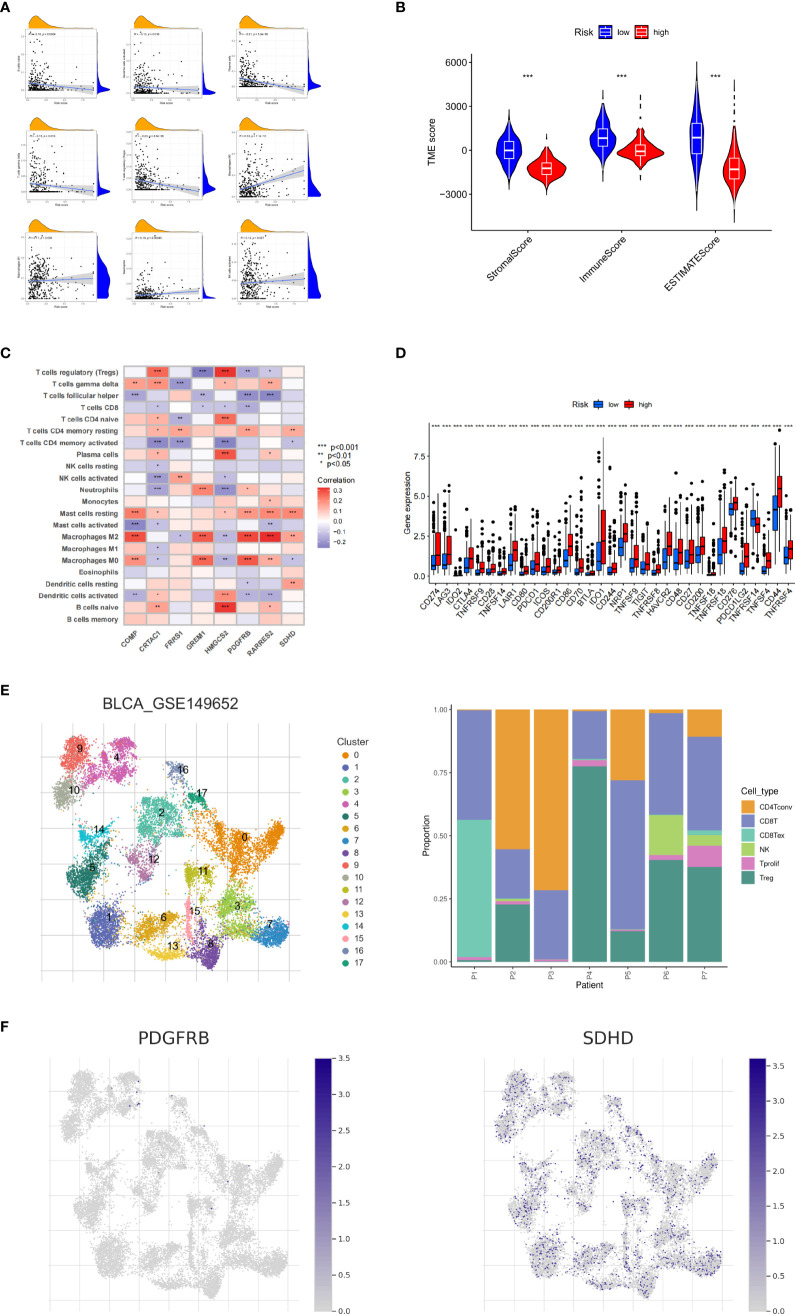
Tumor microenvironment and immune checkpoints for cuproptosis-related gene scores. **(A)** Correlation between cuproptosis-related gene scores (CRG_score) and immune cell type. **(B)** Correlation between CRG_score and immune and stromal scores. **(C)** Correlation between the abundance of immune cell infiltrates and cuproptosis-related genes. **(D)** Differences in immune checkpoint expression among different risk groups. **(E)** Information of BLCA_GSE149652. **(F)** The expression level of SDHD and PDGFRB in TME in single cells. *P < 0.05, **P < 0.01, ***P < 0.001

Next, we explored the correlation between CRG_score and immune cell infiltration characteristics in the TME. The scatter plot results showed that the CRG_score was negatively correlated with naive B cells, activated dendritic cells, plasma cells, γδ T cells, and regulatory T cells and positively correlated with M0 and M1 macrophages, neutrophils, and activated NK cells (**Figure 6A**). The high-risk group had significantly lower StromalScore, ImmuneScore, and ESTIMATE scores than the low-risk group (**Figure 6B**). We also analyzed the relationship between the eight key genes and immune cell abundance, and all were significantly associated with the abundance of multiple infiltrating immune cells (**Figure 6C**). Furthermore, we investigated the association between the immune checkpoints and our risk model. **Figure 6D** shows that 33 immune checkpoints are differentially expressed in the two groups, including PD-1 and PD-L1. These results indicated that the cuproptosis score was closely related to TME. In addition, we analyzed the expression of cuproptosis-related genes in the single-cell immune microenvironment through the BLCA_GSE149652 dataset in the TISCH database. The BLCA_GSE149652 data set divides cells into 17 clusters and contains 6 types of immune cells (**Figure 6E**). The results of the analysis showed that SDHD was expressed in a variety of immune cells, while PDGFRB was less expressed, and none of the other genes were detected (**Figure 6F**).

### 3.8 Validation of prognostic signature gene expression

**Figure 7:**
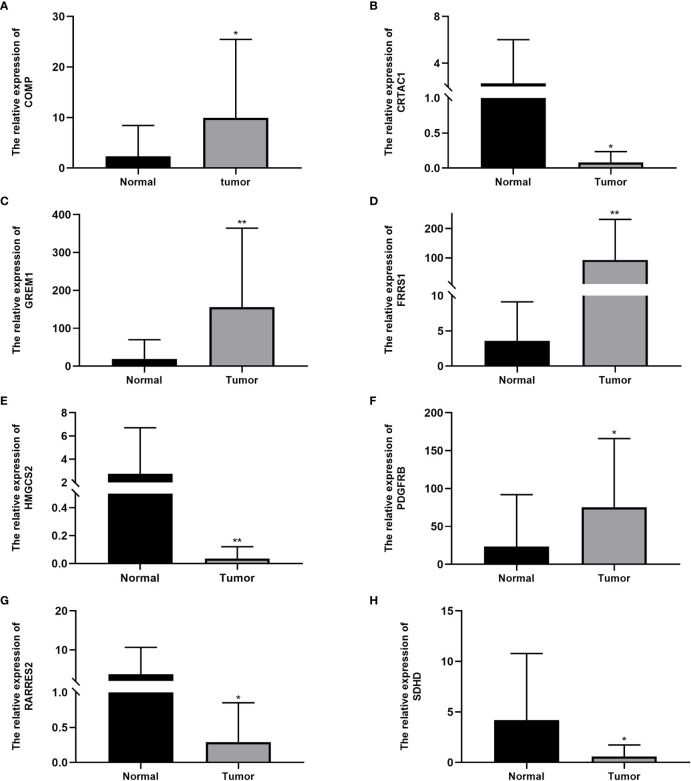
Expression levels of cuproptosis-related genes in bladder cancer tissues and normal tissues: **(A)** *COMP*, **(B)** *CRTAC1*, **(C)** *GREM1*, **(D)** *FRRS1*, **(E)** *HMGCS2*, **(F)** *PDGFRB*, **(G)** *RARRES2*, and **(H)** *SDHD*. Data are presented as the mean ± standard deviation. *P < 0.05, **P < 0.01.

The qRT-PCR analysis of 20 pairs of BC and adjacent non-tumor tissues showed that the expression of *PDGFRB, COMP, GREM1*, and *FRRS1* was higher in BC tissues than in non-tumor tissues. *SDHD, RARRES2, CRTAC1*, and *HMGCS2* were lowly expressed in BC tissues (**Figures 7A–H**).

### 3.9 Correlation of tumor mutational burden and drug sensitivity

**Figure 8:**
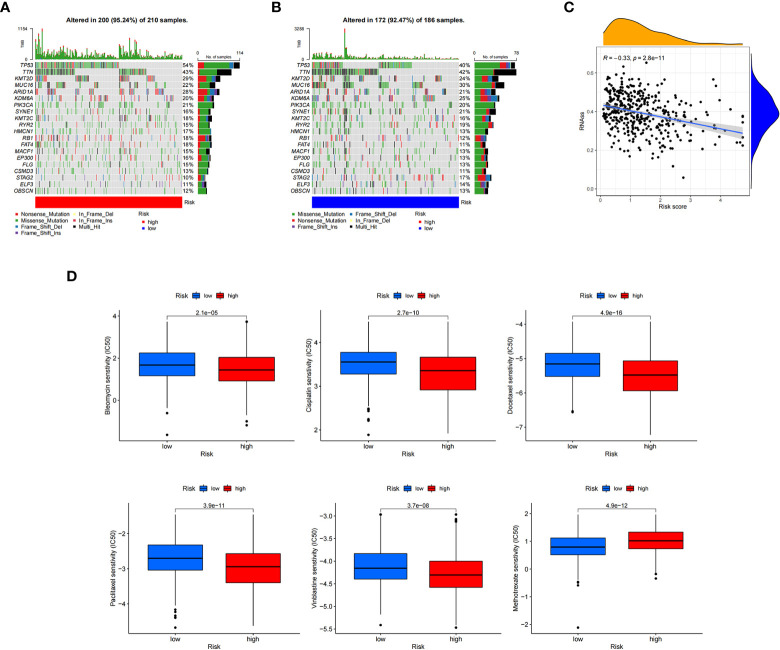
Calculating tumor mutational burden and chemosensitivity by cuproptosis-related gene scores. **(A, B)** Waterfall plot of somatic mutation signature of high- and low-risk groups by cuproptosis-related gene score (CRG_score). **(C)** Relationship between CRG_score and cancer stem cell index. **(D)** Relationship between CRG_score and chemotherapeutic drug sensitivity.

To further explore the somatic mutations among BC cuproptosis score-related subtypes, we analyzed the data from the TCGA database. Most samples in the high- and low-risk groups had somatic mutations. The five genes with the highest frequency were *TP53, TTN, KMT2D, MUC16*, and *ARID1A*. *TP53*, *KMT2D*, and *ARID1A* showed higher mutation frequencies in the high cuproptosis score group, while the opposite was observed for other genes, such as *MUC16*, *SYNE*, and RYR2, in the low-risk group compared with those in the high-risk group (**Figures 8A, B**). We also evaluated the linear relationship between the CRG_score and cancer stem cell (CSC) index. The cuproptosis score was negatively correlated with the CSC index (R = −0.33, P < 0.001). BC cells with lower CRG_scores had a lower degree of cell differentiation and more abundant stem cell properties (**Figure 8C**). In addition, we investigated the relationship between CRG_score and the efficacy of commonly used chemotherapies and targeted therapies for BC (**Figure 8D**). The results showed that the CRG_score was associated with the response to 84 drugs. Among the clear first-line and second-line treatment drugs for BC, patients with low CRG_scores were associated with lower IC50 values of methotrexate, while patients with high CRG_scores had lower IC50 values for vinblastine, docetaxel, bleomycin, cisplatin, and paclitaxel. These results suggest that the CRG_score can be used as a predictor of drug response in BC.

## 4 Discussion

Although treatment methods are constantly developing, the overall survival rate of patients with BC is still not ideal. Therefore, developing new and more effective molecular subtypes, more accurate prognostic evaluations, and personalized treatments are crucial to improving the survival rate of patients with BC. Copper dysregulation has been found in many tumors (31), and many studies have confirmed that copper is related to the occurrence and development of tumors, TME, and immunotherapy responses (32, 33). A new concept of non-apoptotic cell death, cuproptosis (34), has also been described recently, but its effects on BC remain unknown. Therefore, we investigated the expression and mutation changes of cuproptosis-related genes in BC, explored the impact of CRGs on patient prognosis and TME, and established a cuproptosis-related risk score system to predict patient prognosis and guide treatment selection.

The TME refers to the local environment of the tumor where immune and stromal cells, including mesenchymal and endothelial cells, are the two major non-tumor components. There are also secreted products, such as cytokines and chemokines, and the extracellular matrix, consisting of collagen, laminin, and proteoglycans, which constitute the non-cell components (35–37). Tumor cells can reshape the TME, which in turn can further affect the behavior, differentiation level, and tumor cell state, thus affecting patient prognosis (36). Programmed cell death can regulate the immune-related TME (38) and refers to the active and orderly manner of cell death determined by genes and reactive oxygen species level, and these processes are apoptosis, necroptosis (39, 40), pyroptosis (41), and ferroptosis (42). Programmed cell death can affect the tumor immune microenvironment by releasing cell contents, regulating effector cells, or enriching immune cells (43). As a newly discovered mode of programmed cell death, cuproptosis is characterized by the direct binding of copper ions to the lipoacylated components of the TCA cycle in mitochondrial respiration, which leads to the aggregation of lipoacylated proteins and the downregulation of iron-sulfur cluster proteins (44). Lipoacylated components and iron-sulfur cluster proteins are widely present in cells, and they may be targets for tumor therapy.

In this study, we identified and validated two cuproptosis subtypes in BC, where Subtype A has a high level of CRG expression and low levels of immune cell infiltration and highly expresses metabolic pathways. Subtype B CRGs have lower expression levels, but a higher number of immune cell infiltrates, showing characteristics related to the metastatic pathway, which is a low metabolic and high immune infiltration subtype. The two subtypes also showed differences in gene expression and clinical features. We observed that subtype A had better overall survival than subtype B, but there was no evident trend in clinical features. In terms of immunity, subtype B showed higher immune cell infiltration.

We constructed a prognostic model based on the cuproptosis subtype-related prognostic DEGs by LASSO and multivariate COX regression analysis, which consisted of eight gene predictors (*PDGFRB, COMP, GREM1, FRRS1, SDHD, RARRES2, CRTAC1*, and *HMGCS2*). Black et al. found that *PDGFRB* co-expressed with *EGRF*, exerted a combinatorial effect to induce resistance to EGFR-targeted therapy, and increased the tumorigenicity and invasiveness of bladder tumors (45). This gene encodes for non-collagenous extracellular matrix proteins, which participate in tumorigenesis, cancer development, and the epithelial-mesenchymal transition. The *COMP* gene predictor increases colon cancer cell proliferation and may be a predictor in BC. *GREM1* is a BMP antagonist expressed in bone marrow mesenchymal stem cells and fibroblasts. *GREM1* can inhibit tumor cell proliferation, migration, and invasion in various cancers. *FPRS1*, an enzyme that reduces ferric to ferrous iron prior to its transport to the cytoplasm, is a potential tumor regulator. It is a key enzyme located in the inner mitochondrial membrane and is involved in the TCA cycle and electron transport chain. *SDHD*, which encodes SDH, has a role in tumor suppression. Abnormal *RARRES2* expression has been detected in tumor cells. *RARRES2* acts as a chemokine through recruitment and plays an important role in tumor immunity. *CRTAC1* is an extracellular matrix protein-coding gene in cartilage and can promote cell proliferation, migration, and extracellular matrix remodeling. Abnormal *CRTAC1* expression is related to the occurrence and development of BC. *HMGCS2* is a mitochondrial enzyme involved in the ketogenic pathway and can be a tumor suppressor by altering lipid metabolism and adjusting cholesterol synthesis. The expression of these predictors was further confirmed by qRT-PCR in paired BC and normal bladder tissues. The relationship between these predictors and BC has not been reported; therefore, this study provides a new direction for developing prediction and therapeutic targets for BC.

We also studied the cuproptosis score’s effect on immune infiltration. There was a clear correlation between this score and immune cells, immune score, and immune checkpoints. Tumor-associated immune cells within the TME play an important role in the occurrence and development of tumors through pro- or anti-tumor mechanisms. Normally, immune cells recognize and destroy nascent tumor cells; however, because of the unstable cancer genotype, these immune cells may become tumor-associated or supporters, thus promoting tumor survival. In the high-risk group, we found naive B cells, activated dendritic cells, plasma cells, γδ T cells, and regulatory T cells were decreased, but M0 and M1 macrophages, neutrophils, and NK cells were increased. In addition, the CRG_score was positively correlated with the StromalScore, ImmuneScore, and ESTIMATEScore. We also demonstrated that high- and low-score groups of CRG_score have different immune checkpoint pathways activated. We also indicated the optimal drug therapy regimens for treating BC. We measured the sensitivity of patients to different drugs in the CRG_score group. According to IC50 values, vincristine, docetaxel, bleomycin, cisplatin, and paclitaxel showed a better response in the high-risk CRG_score group, whereas methotrexate showed a better response in the low-risk group. These results provide a basis for the individualized treatment selection for patients with BC.

However, our study has some limitations. The data were from public databases, and there was a selection bias. Additionally, our analysis was retrospective and needed to be confirmed by larger prospective studies, and further *in vivo* and *in vitro* experiments are required to confirm our results. Lastly, gene interactions are incredibly complex, and we should continue to explore new and practical modeling methods in future studies.

## Data availability statement

Publicly available datasets were analyzed in this study. This data can be found here: https://portal.gdc.cancer.gov;  https://www.ncbi.nlm.nih.gov/geo/query/acc.cgi?acc=GSE13507;  https://www.ncbi.nlm.nih.gov/geo/query/acc.cgi?acc=GSE31684;  https://www.ncbi.nlm.nih.gov/geo/query/acc.cgi?acc=GSE32894.

## Ethics statement

The studies involving human participants were reviewed and approved by the Ethics Committee of the Fourth Affiliated Hospital of Harbin Medical University. The patients/participants provided their written informed consent to participate in this study.

## Author contributions

Conceptualization, HW and XY. Methodology, HW. Software, HW and JL. Validation, HW and XZ. Formal analysis, HW. Investigation, HW. Resources, HW. Data curation, XY. Writing—original draft preparation, HW. Writing—review and editing, HW. Visualization, HW. Supervision, XY. All authors have read and agreed to the published version of the manuscript.
