# Supplementary material for: Comprehensive analysis of cuproptosis-related genes on bladder cancer prognosis, tumor microenvironment invasion, and drug sensitivity
Source: Front Oncol. 2023 Feb 21;13:1116305. doi: 10.3389/fonc.2023.1116305 (PMC9989218; doi:10.3389/fonc.2023.1116305)
Supplement: Supplementary file 1 [file Table_1.pdf]

Table S1 List of 13 cuproptosis-related genes

**Gene name**
FDX1
LIPT1
LIAS
DLD
DBT
GCSH
DLST
DLAT
PDHA1
PDHB
SLC31A1
ATP7A
ATP7B
