# Supplementary material for: Comprehensive analysis of cuproptosis-related genes on bladder cancer prognosis, tumor microenvironment invasion, and drug sensitivity
Source: Front Oncol. 2023 Feb 21;13:1116305. doi: 10.3389/fonc.2023.1116305 (PMC9989218; doi:10.3389/fonc.2023.1116305)
Supplement: Supplementary file 2 [file Table_2.pdf]

Table S2 List of 126 DEGs associated with cuproptosis subtypes

Gene name
DLD
DLAT
SDHD
ACADM
G3BP2
TFB2M
ISOC1
SCO1
TSPYL1
NUDT21
CCNG1
LANCL1
PRDX3
RCN2
SLC39A6
IARS2
PRMT6
NMD3
LMBRD1
BCKDHB
SYPL1
SCOC
ETFA
CD2AP
PLS1
CAT
LGR4
SKAP2
TMEM123
TMEM14A
PGM2
DSG2
GCLC
PGK1
DDX21
TOP2B
MANSC1
IDH1
ALDH5A1
RDX

YEATS4
WEE1
MAP7
ABCC4
PLA2G4A
TSPAN13
EHF
ILDR1
THY1
TSPAN12
ENPP4
SLAIN1
CYB5A
ANKRD50
GNAI1
PTPN13
MGST1
CRYZ
PCOLCE
HS6ST2
SLC44A3
UGT1A6
AEBP1
CXADR
SGPP2
PIR
TAGLN
BGN
CD248
BTG2
ODC1
FOXA1
TPM2
F2RL1
PDGFRB
GAS6
COL16A1
ITGA5
GSPT2
MT2A
MYL9
RARRES2
GPR160

MAL2
COL6A2
CNTN3
COL1A1
METTL7A
CPXM1
COL7A1
NNMT
CTSK
IL32
BMP3
MAOA
COL5A1
ISLR
ACTG2
GZMB
RNF128
PPP1R3C
COMP
SFRP4
INA
CTHRC1
GRHL3
GREM1
CNN1
SFRP2
AKR1C3
CCL19
AGR2
MMP9
FOXQ1
S100A8
DES
CRTAC1
BHMT
S100A9
ANXA10
CYP4B1
CLCA4
UPK1B
HMGCS2
FRRS1
KRT14
