# Supplementary material for: Comprehensive analysis of cuproptosis-related genes on bladder cancer prognosis, tumor microenvironment invasion, and drug sensitivity
Source: Front Oncol. 2023 Feb 21;13:1116305. doi: 10.3389/fonc.2023.1116305 (PMC9989218; doi:10.3389/fonc.2023.1116305)
Supplement: Supplementary file 3 [file Table_3.pdf]

Table S3 List of 82 DEGs related to prognosis

**Gene name**
SDHD
ISOC1
SCO1
CCNG1
IARS2
LMBRD1
BCKDHB
CD2AP
PLS1
CAT
GCLC
MANSC1
IDH1
ALDH5A1
MAP7
ABCC4
PLA2G4A
ILDR1
THY1
TSPAN12
SLAIN1
CYB5A
ANKRD50
PTPN13
PCOLCE
HS6ST2
SLC44A3
AEBP1
CXADR
SGPP2
PIR
TAGLN
BGN
CD248
BTG2

FOXA1
TPM2
PDGFRB
GAS6
COL16A1
ITGA5
MT2A
MYL9
RARRES2
GPR160
COL6A2
CNTN3
COL1A1
METTL7A
CPXM1
COL7A1
NNMT
CTSK
BMP3
MAOA
COL5A1
ISLR
ACTG2
RNF128
PPP1R3C
COMP
SFRP4
INA
CTHRC1
GRHL3
GREM1
CNN1
SFRP2
AGR2
MMP9
FOXQ1
S100A8
DES

CRTAC1
BHMT
S100A9
ANXA10
CYP4B1
CLCA4
HMGCS2
FRRS1
KRT14
