# Supplementary material for: Comprehensive analysis of cuproptosis-related genes on bladder cancer prognosis, tumor microenvironment invasion, and drug sensitivity
Source: Front Oncol. 2023 Feb 21;13:1116305. doi: 10.3389/fonc.2023.1116305 (PMC9989218; doi:10.3389/fonc.2023.1116305)
Supplement: Supplementary file 4 [file Table_4.pdf]

Table S4 Primer sequences of genes

Gene name		Sequence(5'-3')
SDHD	F	CTTCAGGACCGACCTATCCC
	R	AGCCCAGCAAAGGTTAAAGC
PDGFRB	F	AGACTGTTGGGCGAAGGTTA
	R	CTGCACGGCAGTATAGAGGA
RARRES2	F	CAGGAGACCAGTGTGGAGAG
	R	CTCAGAGCCCAGTTTGATGC
COMP	F	CCAGGACGACTTTGATGCAG
	R	TTGTCTGCACGATCTCCCTT
GREM1	F	CGCTTAAGCAGACCATCCAC
	R	AAGGAACCTTCCTCCTTCCG
CRTAC1	F	GAAGGATGAAGCCAGCAGTG
	R	GGGTAGAGGATCTCCAGCAC
HMGCS2	F	ACCCTTCACCCTTGACGATT
	R	TGGTTTTGTGTGTCACTGCTG
FRRS1	F	TCTGAAGGTTCATGGTGCCT
	R	AACCAAGCTGCTTCACCAAG
